# Crowdedness Mediates the Effect of Social Identification on Positive Emotion in a Crowd: A Survey of Two Crowd Events

**DOI:** 10.1371/journal.pone.0078983

**Published:** 2013-11-13

**Authors:** David Novelli, John Drury, Stephen Reicher, Clifford Stott

**Affiliations:** 1 Department of Psychology, University of Hertfordshire, Hatfield, United Kingdom; 2 School of Psychology, University of Sussex, Brighton, United Kingdom; 3 School of Psychology and Neuroscience, University of St Andrews, St Andrews, Scotland, United Kingdom; 4 School of Law, University of Leeds, Leeds, United Kingdom; University of Leicester, United Kingdom

## Abstract

Exposure to crowding is said to be aversive, yet people also seek out and enjoy crowded situations. We surveyed participants at two crowd events to test the prediction of self-categorization theory that variable emotional responses to crowding are a function of social identification with the crowd. In data collected from participants who attended a crowded outdoor music event (n = 48), identification with the crowd predicted feeling less crowded; and there was an indirect effect of identification with the crowd on positive emotion through feeling less crowded. Identification with the crowd also moderated the relation between feeling less crowded and positive emotion. In data collected at a demonstration march (n = 112), identification with the crowd predicted central (most dense) location in the crowd; and there was an indirect effect of identification with the crowd on positive emotion through central location in the crowd. Positive emotion in the crowd also increased over the duration of the crowd event. These findings are in line with the predictions of self-categorization theory. They are inconsistent with approaches that suggest that crowding is inherently aversive; and they cannot easily be explained through the concept of ‘personal space’.

## Introduction

Exposure to crowding – that is, close physical proximity in a crowd – has been shown to be detrimental to human experience and wellbeing [1]–[3]. However, as far back as the 1970s, there has been evidence that has served to question the inevitability of negative outcomes of crowding, and to suggest instead that crowding can be experienced positively [4]–[6]. Many events which draw large crowds - such as festivals, concerts, and football matches - can be scenes of collective joy, so such positive experience is not surprising. What is surprising, perhaps, is that, with few exceptions [7]–[9], positive emotional responses associated with being in dense crowds have remained under-investigated by psychologists.

Most notable of the attempts to explain variable responses to crowding is the concept of ‘personal space’ [10]–[12]. This approach suggests that, when a perceiver feels that their zone of ‘personal space’ is intruded upon, they will experience negative arousal which they attribute to the physical encroachment of the other people present. The strength of this approach as an account of variable responses to crowding is that it considers the physical and social relations between the perceiver and the others co-present in the crowd, rather than simply focussing on individual personality variations [4] or on complex interactions between several intervening factors [13].

However, there are a number of limitations of the ‘personal space’ approach. First, it can only account for those situations where close proximity is avoided or experienced negatively (i.e., as uncomfortable or stressful) – such as on a packed commuter train. It does not provide an explanation as to why people actually seek out and enjoy close proximity to others - as might be the case on a packed football terrace. Second, the ‘personal space’ approach relies on a long list of moderating variables, such as culture [14], age [15], gender role [16], ‘race’ [17], clinical condition [18], or the location of an interaction [19], to explain why and when close proximity might be perceived as an intrusion. In doing so, the approach cannot provide a satisfactory explanation of intra-cultural, intra-individual, or within-location variability in responses to crowding.

An alternative to both the traditional ‘crowding’ approach and the concept of ‘personal space’ can be proposed based on self-categorization theory [20], [21]. Self-categorization theory suggests that we have multiple selves or social identities, which vary in salience depending on context. In this account, our desire for, and response to, close physical proximity is a function of our *psychological* proximity to those people with whom we are co-located. When we are with those perceived as an extension of self (i.e., other in-group members), we might enjoy their physical proximity and therefore seek to be close to them. However, when we are with those perceived as ‘other’ (i.e., other individuals when we self-categorize in terms of our personal identity, or out-group members when self-categorized in terms of a social identity) we instead seek a spatial distance that reflects our psychological distance; or, if any close physical proximity is unavoidable, we might experience the incongruence between our psychological and physical distance as stressful or unpleasant.

The only test of this hypothesized relation between identity and crowding that has been carried out that we are aware of is that by Novelli et al. [22], which experimentally manipulated group identities and found that participants’ chosen seating location was closer to people in the same social group than to those in a different group, even though the two groups were bogus and had no prior history. However, this research was conducted in a laboratory environment; it focussed on one-to-one proximity; and the participants only anticipated interaction (as opposed to experiencing the close proximity of others). It also did not actually measure emotional responses, only behaviour. What is still required, therefore, is to test the prediction of self-categorization theory of the relation between identity, location and emotional experience in an actual crowd setting with appropriate measures. The research described in this paper therefore aimed to validate the laboratory findings of Novelli et al. [22] by demonstrating in the field that relevant social identification increases positive emotional responses in situations of crowdedness.

### Overview

We carried out two questionnaire studies. In the first, we surveyed participants who had been to an outdoor music event that was overcrowded on objective measures. In the second, in an improved design, we surveyed participants as they took part in a protest demonstration. In each case, we took measures of social identification (with the crowd), crowdedness, and positive emotional responses. We expected, and found, that identification with the crowd affected the extent to which participants felt crowded and their central location in the crowd, and that these measures of crowdedness mediated the effect of identification with the crowd on positive emotion.

## Study 1

We carried out a survey amongst a sample of people who had attended a free music event featuring the dance DJ Fatboy Slim, which took place in Brighton, UK, on July 13^th^ 2002. The event organizers planned for a crowd of approximately 65,000 people. The Safety Manual for the event stated that the site was 50,605 m^2^ in size, and therefore allowed for 0.5 m^2^ per person in a standing crowd [23]. However, most estimates put the size of the crowd that day at around 250,000 [24], [25], giving only 0.2 m^2^ of space per person. Prima facie, therefore, this was a very crowded event. We took measures of participants’ feelings of crowdedness, as well as their identification with the crowd, and their positive emotion at the event. We had two hypotheses. The first was that crowdedness would mediate the relation between identification with the crowd and positive emotion; specifically, identification with the crowd would predict feeling less crowded, which would in turn predict positive emotion. The second hypothesis was that identification with the crowd would moderate the relation between feeling crowded and positive emotion; that is, any negative relation between feeling crowded and positive emotion would not apply to those scoring high on identification with the crowd.

### Method

We developed a questionnaire which was hosted online via the Bristol Online Survey website [26] in March 2011.

#### Participants

Forty-eight participants completed the questionnaire, for a fee of £5 each. Ages ranged from 25–59 (*M* age = 35.87, *SD* = 7.50). Thirty were female and 18 were male. Twenty of the participants lived in Brighton and/or the surrounding area at the time of the event, whereas 24 were living in other parts of the UK. Four participants did not disclose residential information. One sample *t*-tests (comparing mean scores with the scale mid-point of 4) revealed that at the time of the event, the sample were fans of the DJ Fatboy Slim (*M* = 5.54, *SD* = 1.15), *t*(47) = 9.31, *p* = .001, familiar with the dance music scene (*M* = 6.10, *SD* = 1.17), *t*(47) = 12.45, *p* = .001, and regularly attended nightclubs and dance music parties (*M* = 5.92, *SD* = 1.43), *t*(47) = 9.31, *p* = .001.

#### Measures

All items were measured on 7-point Likert scales anchored by 1 (‘disagree strongly’) and 7 (‘agree strongly’).

***Feeling too crowded.*** There were four items in the questionnaire on feeling too crowded: ‘I would have liked to have been further away from the other crowd members at the Fatboy Slim beach party in 2002’ ‘I felt crowded at the Fatboy Slim beach party in 2002’, ‘I enjoyed being in close proximity to other crowd members at the Fatboy Slim beach party in 2002’ (reversed), and ‘I felt uncomfortable with the close physical proximity of the other crowd members at the Fatboy Slim beach party in 2002’ (α = .78).

***Social identification.*** There were three items on identification with the crowd (based on those in Doosje et al. [27], [28]):‘I identified with the other crowd members at the 2002 Fatboy Slim beach party’, ‘I am like the other people who were at the 2002 Fatboy Slim beach party’, and ‘I felt strong ties with the other people who were at the 2002 Fatboy Slim beach party (α = .84).

***Positive emotion.*** The questionnaire contained three items on positive emotion: ‘I felt joyful at the 2002 Fatboy Slim beach party’, I felt excited at the 2002 Fatboy Slim beach party’, and ‘I felt cheerful at the 2002 Fatboy Slim beach party’ (α = .98).

#### Ethics statement

Ethical clearance was given for this study by the University of Sussex School of Life Sciences Ethics Committee in October 2010. All participants provided their written informed consent prior to participation.

### Results

The mean score for feeling too crowded was 3.91 (*SD* = 1.17), and results of a one-sample *t*-test suggested that this was not significantly different from the scale mid-point of 4, *t*(47) = −.51, *p* = .61. This suggests that, overall, participants were neutral regarding whether they felt too crowded.

**Figure 1:**
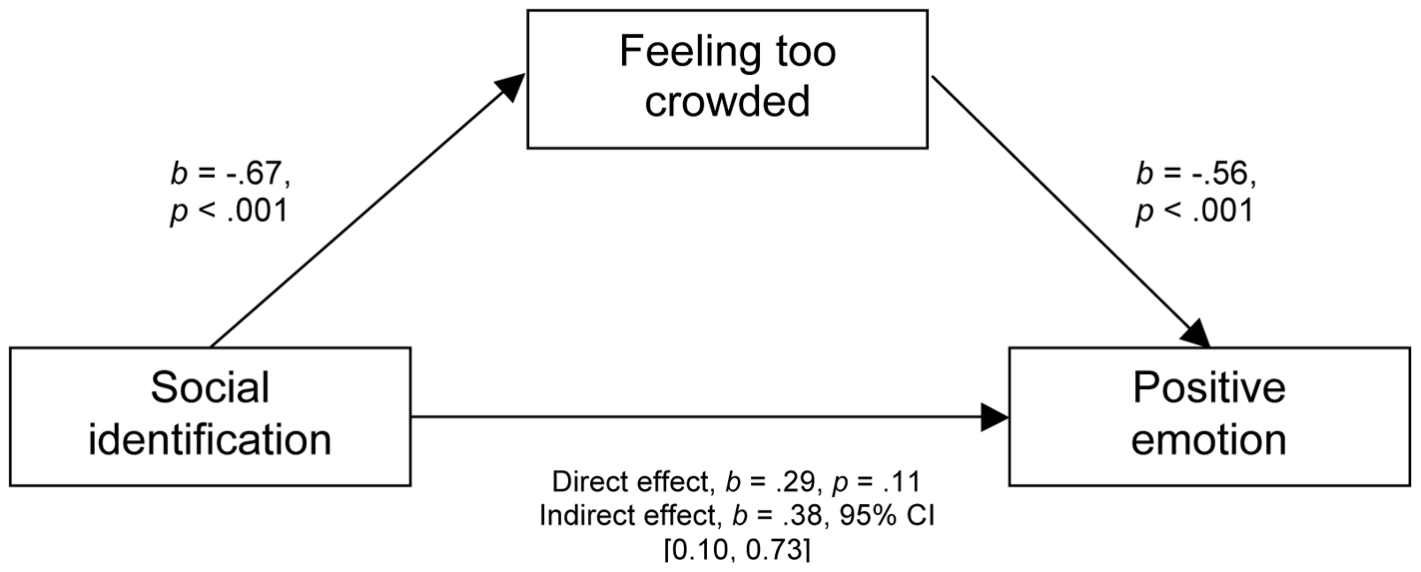
Model of social identification as predictor of positive emotion, mediated by feeling too crowded. The confidence interval for the indirect effect is a BCa bootstrapped CI based on 1000 samples.

As expected, identification with the crowd (negatively) predicted feeling too crowded, *β* = −.52, *p*<.001, *R^2^* = .28; that is, the more that people identified with the crowd the less they reported feeling too crowded. We conducted mediation analyses based on 1,000 bootstrap samples using the Process tool devised by Hayes [29]. As expected, there was a significant indirect effect of identification with the crowd on positive emotion through feeling too crowded, *b* = 0.38, 95% BCa CI [0.10, 0.73], κ^2^ = .29, which represents a small to medium effect [30] - see Figure 1. In other words, crowdedness mediated the effect of identification with the crowd on positive emotion. A reverse mediation model, in which crowdedness mediated the effect of positive emotion on identification with the crowd, was also significant, with a slightly reduced effect size, *b* = −0.61, 95% BCa CI [0.04, 0.39], κ^2^ = .23.

We conducted a moderation analysis, again using the Hayes Process tool [29], [31]. As expected, identification with the crowd moderated the effect of feeling too crowded on positive emotion – see Table 1.

**Table 1:** Linear model of predictors of positive emotion, with 95% confidence intervals.

	*b*	*SE B*	*t*	*p*
Constant	6.24 [5.91, 6.58]	.17	37.55	<.001
Identification with the crowd (centred)	.38 [−.003, .765]	.19	2.00	.05
Feeling too crowded (centred)	−.43 [−.78, −.09]	.17	−2.51	.02
Identification with the crowd × Feeling too crowded	.26 [.01, .51]	.13	2.08	.04

Note: R^2^ = .55.

**Figure 2:**
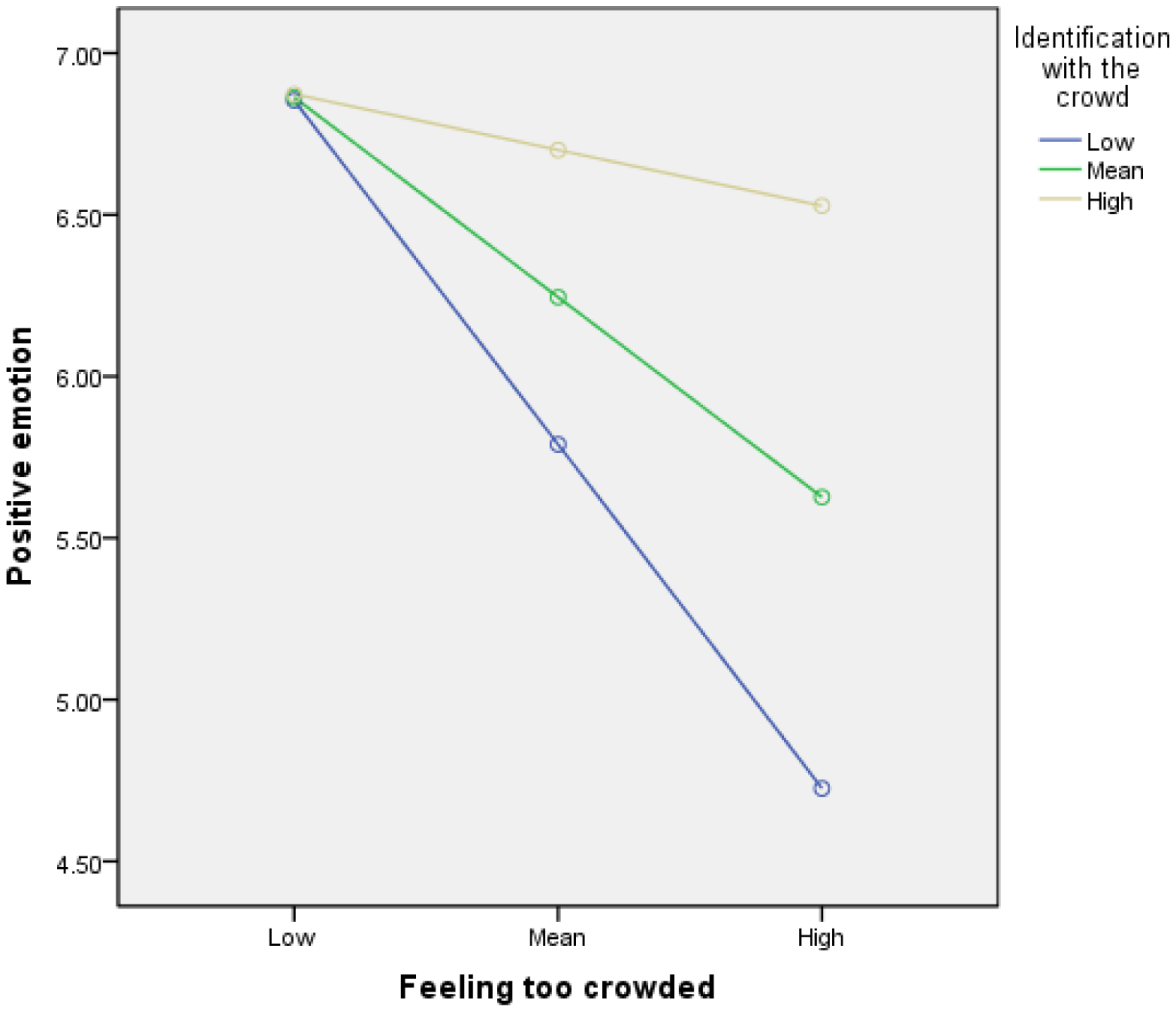
Simple slopes equations of the regression of positive emotion on feeling too crowded at three levels of identification with the crowd. Note: Low = 1 *SD* below the centred mean; high = 1 *SD* above the centred mean.

Simple slopes analysis (Figure 2) shows that, at low levels of identification with the crowd (blue line), as participants felt more crowded they were less likely to report positive emotion, *b* = −.75, 95% BCa CI [−1.17, −0.21], *t* = −3.52, *p* = .001. But at high levels of identification with the crowd (beige line), as participants feltl more crowded this had no significant effect on positive emotion, which remained high, *b* = −.12, 95% BCa CI [−0.61, 0.37], *t* = −0.49, *p* = .37.

### Discussion

Overall, the participants from an outdoor music event we surveyed did not report feeling too crowded, despite the fact that the event was very crowded on objective measures. However, in line with previous research on crowding, the more that people reported feeling crowded, the less positive emotion they reported. Yet, as we hypothesized and as shown by the mediation analysis, identification with the crowd predicted feeling less crowded, which in turn explained their positive emotions. Moreover, the moderation analysis showed that whereas low identifiers became more negative emotionally as they felt more crowded, high identifiers did not.

Identification operated as a predictor in the first analysis and as a moderator in the second analysis. Both predictions made sense theoretically, as did their combination: identification with the crowd should *both* predict feelings about crowdedness and position emotions, *and* whether or not crowdedness is enjoyed. As well as theoretical reasons, there are established statistical justifications for using the same variables in both mediation and moderation tests [32].

The findings from Study 1 are in line with the suggestion, based on self-categorization theory [20], [21], that identification with a crowd is one possible reason why people can sometimes enjoy being in the midst of dense crowds [22]. However, there are two key limitations of the study. The first is that the survey data was collected some time after the event, meaning that memories of the experience may have faded (though there is evidence that involvement in ‘extreme’ events enhances our recall of them [33]). A better design would entail collecting data *during* the crowd event. The second limitation of the study is that the mediator measure relating to ‘crowdedness’ was the participants’ reports that they wanted more space. It was not a measure of being within a crowded location. It would be preferable to use a measure of location in the crowd without an evaluative component to test its mediating effects on the relationship between identification with the crowd and positive emotion. These, therefore, were the aims of Study 2.

## Study 2

We conducted a cross-sectional survey at a demonstration march and rally, which took place in central London on November 3^rd^ 2007. The event was organised by UNISON (Britain’s largest public sector union) and was intended as a show of support for the National Health Service (NHS) in the context of threats of privatization and cuts. It was estimated by UNISON that 7,000 people took part [34]. The demonstration took place in three phases: (1) assembly at Temple Place, Victoria Embankment, from 11.00 am until 12.30 pm; (2) a mile-long march along Victoria Embankment and through Westminster; and (3) a rally, which took place in Trafalgar Square from 1.30 pm onwards. Data collection took place at the assembly point (henceforth phase 1) and the rally (phase 2). Pictures of the event suggest that the level of density varied (for example [35]), but that it was usually greatest in central locations of the march (we estimate roughly 0.3 m^2^ of space per person), rather than at the edges of the crowd, where people tended to be more spread out.

We predicted that participants in a more central (and hence more crowded) location in the crowd would report more positive emotion, contrary to the density-aversion hypothesis, and that identification with the demonstration crowd would be associated with a more central location in the crowd (and hence closer proximity to fellow crowd members). Second, we predicted mediation, in that there would be an indirect effect of social identification on positive emotion through location in the crowd: that is, identification with the demonstration crowd would lead to positive emotion because people would be in close physical proximity to those they identified with. Third, following the results of Study 1, we also expected a moderation effect, whereby high identifiers would be the part of the sample reporting most positive emotion in the central location in the crowd. Finally, if positive emotion is an effect of close physical proximity and social identification with the crowd, then we would also expect to find an increase over time in positive emotion (though not necessarily proximity and identification); this then was a fourth prediction, and was therefore the reason that we sampled at two separate phases over the duration of the event.

### Method

#### Participants

Demonstrators were asked to take part in a survey on participant perceptions of demonstrations. In total, 112 agreed to take part (58 female, 54 male). (Ten people declined to complete the questionnaire, but did not provide a reason for doing so.) The ages of the sample ranged from 14 to 67 (*M* = 39.22, *SD* = 13.42). Data were collected from 56 demonstrators during each of the two data collection phases (phase 1∶28 male, 28 female; age range = 14 to 67, *M* = 37, *SD* = 15; phase 2∶26 male, 30 female; age range = 19 to 63, *M = *41, *SD* = 11). Based on photographs taken on the day [35], at both phases the sample appears to be broadly representative in terms of the gender balance and age range of those present. Chi squared tests established that there were no significant differences in gender (χ^2^(1) = 0.04, *p* = .85) or age (χ^2^(40) = 45.67, *p* = .25) between participants sampled at phase 1 and those sampled at phase 2.

#### Measures

The questionnaire items used a Likert response scale, which ranged from 1 (not at all) to 7 (very much).

***Location in the crowd.*** We designed three items to measure participants’ location in the crowd for this study: ‘I am right in the middle of the crowd’, ‘I am away from the middle of the crowd’ (reverse scored), and ‘I am in the thick of things’ (*α = *.73). As research on crowding and ‘personal space’ typically focuses on objective physical proximity in a crowd, we compared participants’ estimates with that of the researchers, on a scale which ranged from 1 (periphery) to 7 (centre). We found a small positive correlation between the researcher and participant estimates of location, *r = *.21, *p = *.03, which provides some evidence to confirm the demonstrators’ own estimates of their location in the crowd.

***Social identification.*** We measured participants’ social identification as NHS supporters on the demonstration using three items [27], [28]: ‘I identify with other people at this NHS demonstration’, ‘I am like other people at this NHS demonstration’, and ‘Being a demonstrator on this march is a reflection of who I am’ (*α = *.80).

***Positive emotion.*** Two items were used: ‘I feel happy’ and ‘I am not enjoying the event’ (reverse scored) (*r* = .32, *p = *.001).

#### Procedure

Four researchers arrived at the assembly point at 11.30 am. We approached demonstrators who were waiting for the march to begin and asked them to fill in a questionnaire. To avoid non-independence of responses, only one member of each group of people within the crowd was asked to participate. Having completed the questionnaire, participants were provided with a written debrief. After one hour of data collection, we marched with the demonstrators until they reached Trafalgar Square. We then repeated the data collection process described above at the rally for one hour.

#### Ethics statement

Ethical clearance was given for this study by the University of Sussex School of Life Sciences Ethics Committee in January 2007. All participants provided their written informed consent prior to participation. All participants were 18 years of age or ever, except one, who was 14 years of age. He was approached with his parents, and they and he gave consent (written) for his participation.

### Results

Table 2 shows that participants’ identification with the crowd was significantly positively correlated with their perceived location in the crowd and with their positive emotion. That is, the more that participants identified, the more they positioned themselves in a central location; and a more central location in the crowd was associated with a more positive emotional response. This pattern of results thus supports the first set of hypotheses.

**Table 2:** Social identification with the crowd, location in the crowd, and positive emotion: Means, standard deviations and correlations.

Variable	*M*	*SD*	1	2	3
1. Social identification	5.96	1.13	–	.26*	.32*
2. Location in the crowd	4.96	1.33		–	.25*
3. Positive emotion	5.74	1.32			–

Note: * = *p*<.01.

**Figure 3:**
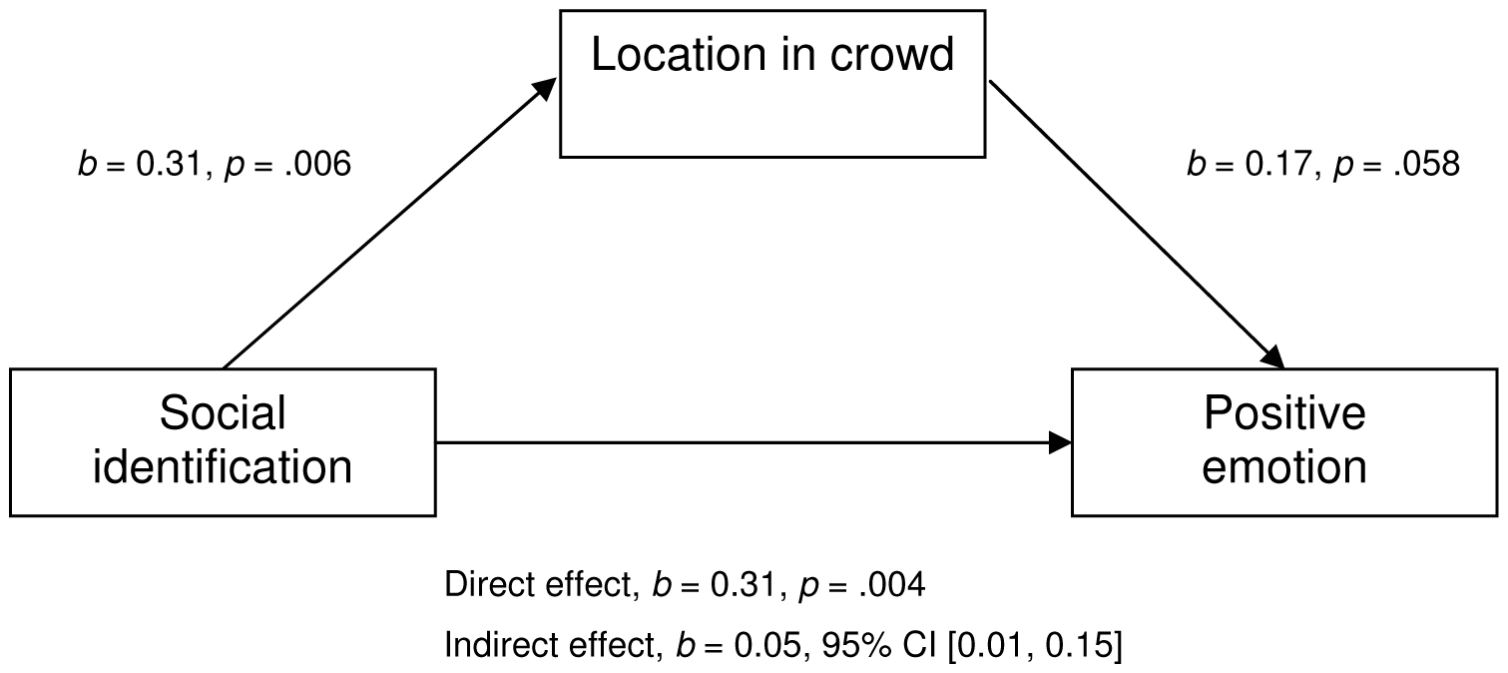
Model of social identification as predictor of positive experience, mediated by location in the crowd. The confidence interval for the indirect effect is a BCa bootstrapped CI based on 1000 samples.

We conducted mediation analyses based on 1,000 bootstrap samples using the Process tool [29]. As expected, there was a significant indirect effect of identification with the crowd on positive emotion through location in the crowd, *b* = 0.05, 95% BCa CI [0.006, 0.146], κ^2^ = .05, which represents a relatively small effect [30] (see Figure 3). That is, central location in the crowd mediated the effect of identification with the crowd on positive emotion. A reverse mediation model, in which central location mediated the effect of positive emotion on identification with the crowd, was also significant, *b* = 0.04, 95% BCa CI [0.005, 0.109], κ^2^ = .04.

**Figure 4:**
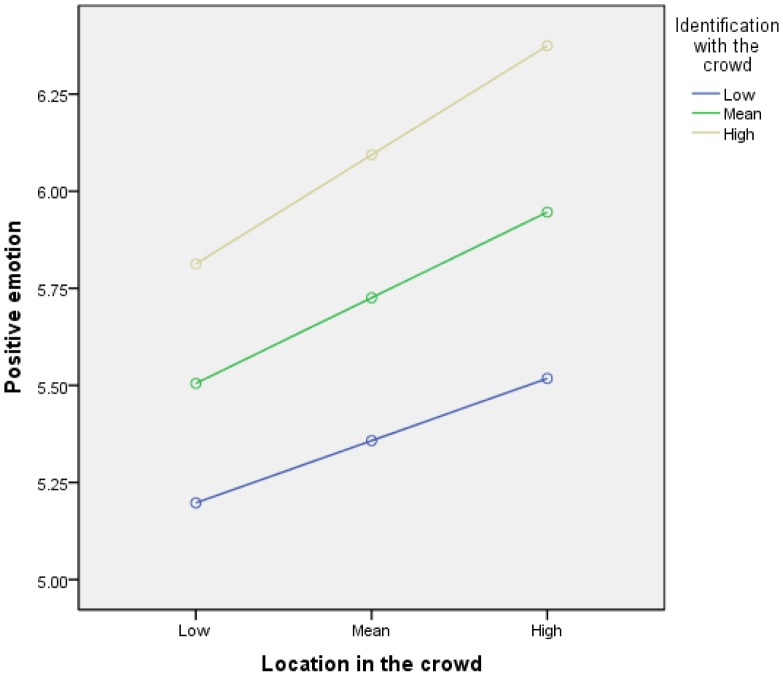
Simple slopes equations of the regression of positive emotion on central location in the crowd at three levels of identification with the crowd. Note: Low = 1 *SD* below the centred mean; high = 1 *SD* above the centred mean.

We conducted a moderation analysis [29], [31]. As Table 3 shows, Identification with the crowd did not significantly moderate the effect of location in the crowd on positive emotion. Figure 4 suggests that the pattern of data is at least partly as expected, in that the highest levels of positive emotion are reported for high identifiers in the most central locations. However, simple slopes analysis showed that, at different levels of identification with the crowd, crowd location had relatively little effect on positive emotion: low identification (blue line on Figure 4) *b* = .12, 95% BCa CI [−0.11, 0.35], *t* = 1.06, *p* = .29; medium identification (green line) *b* = .17, 95% BCa CI [−0.01, 0.34], *t* = 1.89, *p* = .06; high identification (beige line) *b* = .21, 95% BCa CI [−0.05, 0.47], *t* = 1.62, *p* = .11).

**Table 3:** Linear model of predictors of positive emotion, with 95% confidence intervals.

	*b*	*SE B*	*t*	*p*
Constant	5.74 [5.48, 5.98]	.13	45.31	<.001
Identification with the crowd (centred)	.33 [.12, .53]	.10	3.21	.002
Location in the crowd (centred)	.17 [−.01, −.34]	.09	1.89	.06
Identification with the crowd × Location in the crowd	.04 [−.11, .19]	.08	0.53	.59

Note: R^2^ = .13.

Finally, a *t*-test showed that, in line with the fourth prediction, positive emotion increased significantly over time (phase 1 *M* = 5.41, phase 2 *M* = 6.08, *t*(110) = −2.81, *p* = .006). Social identification with the demonstration crowd and perceived centrality of location in the crowd also increased over time, although neither of these changes reached conventional levels of significance (identification: phase 1 *M* = 5.77, phase 2 *M* = 6.14, *t*(110) = −1.72, *p* = .088; centrality of location: phase 1 *M* = 4.73, phase 2 *M* = 5.18, *t*(110) = −1.84, *p* = .069).

### Discussion

In Study 2, a different measure of crowdedness was employed than in Study 1: central location in the crowd. Although significant, the low correlation between researchers’ and participants’ estimates of centrality suggests that location may have a subjective element. Yet the pattern of results was extremely similar to those of Study 1 and largely as predicted. The more that demonstrators identified with the demonstration crowd, the more they were in a central physical location within the crowd, and hence closer in physical proximity to their fellow crowd members in this relatively crowded location. The correlations therefore provide evidence in support of the view that identifying with a crowd, and immersion in a dense crowd, can be associated with positive emotion. Further, the results of the mediation analysis were consistent with self-categorization theory [20], [21], by showing that there was an indirect effect of social identification on positive emotion through perceived location. That is, the more participants identified, the more they also tended to be in a more central physical location, and this physical immersion in the centre of the crowd predicted their more positive emotion. There was also evidence to suggest that these correlations reflected changes over time in people’s experience of being in the crowd: positive emotion increased from phase 1 to phase 2.

These results are therefore in accord with the laboratory findings of Novelli et al. [22], which showed that social identification influences our desire for close physical proximity to ingroup members.

## General Discussion

Across two different types of crowd events, we found an association between crowd identification, measures of crowdedness, and positive emotion. These findings are inconsistent with approaches that emphasize the inherently aversive consequences of crowding. The findings also cannot easily be explained easily by the concept of ‘personal space’, since that concept is premised upon a notion of self as only personal and single, rather than also being collective, multiple and hence variable [22]. Instead, the results are more in line with predictions based on self-categorization theory [20], [21]. Therefore, these findings complement existing laboratory experimental evidence of variable responses to crowded situations [22].

As both of the analyses presented here were essentially correlational, it is of course possible that there are other explanations for the findings. We tested two reverse mediation models, one for each study. While both worked statistically, we are not aware of any theoretical reason to expect positive emotion to lead to identification with a crowd. It may be possible, however, that crowd location influenced social identification (Study 2). The idea that social identification can be affected by physical processes within a crowd is suggested by findings on synchronous co-action in groups, which show that moving together in time can enhance psychological attachment to a group [36]–[38]. Yet this explanation does not seem to make sense of the data from Study 1, where the measure of crowdedness was not location in the crowd but desire for more space, and yet a similar relationship with identification was found.

In conclusion, the present paper has reported two studies that complement each other in showing the psychological conditions under which situations of crowdedness can actually be enjoyed, instead of being inherently aversive. The results suggest that it is those crowds with whom we do not identify that we find uncomfortable, whereas we actively seek central and even dense locations in those crowds which we categorize as ‘us’.
